# Differentiation of *Bacillus pumilus* and *Bacillus safensis* Using MALDI-TOF-MS

**DOI:** 10.1371/journal.pone.0110127

**Published:** 2014-10-14

**Authors:** Raquel Branquinho, Clara Sousa, João Lopes, Manuela E. Pintado, Luísa V. Peixe, Hugo Osório

**Affiliations:** 1 REQUIMTE, Laboratório de Microbiologia, Departamento de Ciências Biológicas, Faculdade de Farmácia, Universidade do Porto, Porto, Portugal; 2 CEB, Centro de Engenharia Biológica, Universidade do Minho, Braga, Portugal; 3 REQUIMTE, Laboratório de Química Aplicada, Departamento de Ciências Químicas, Faculdade de Farmácia, Universidade do Porto, Porto, Portugal; 4 CBQF, Centro de Biotecnologia e Química Fina, Escola Superior de Biotecnologia, Universidade Católica Portuguesa, Porto, Portugal; 5 Departmento de Tecnologia Farmacêutica, Faculdade Farmácia, Universidade de Lisboa, Lisboa, Portugal; 6 IPATIMUP, Instituto de Patologia e Imunologia Molecular da Universidade do Porto, Porto, Portugal; 7 Faculdade de Medicina, Universidade do Porto, Porto, Portugal; INRA Clermont-Ferrand Research Center, France

## Abstract

Matrix-assisted laser desorption/ionization time-of-flight mass spectrometry (MALDI-TOF-MS) despite being increasingly used as a method for microbial identification, still present limitations in which concerns the differentiation of closely related species. *Bacillus pumillus* and *Bacillus safensis,* are species of biotechnological and pharmaceutical significance, difficult to differentiate by conventional methodologies. In this study, using a well-characterized collection of *B. pumillus* and *B. safensis* isolates, we demonstrated the suitability of MALDI-TOF-MS combined with chemometrics to accurately and rapidly identify them. Moreover, characteristic species-specific ion masses were tentatively assigned, using UniProtKB/Swiss-Prot and UniProtKB/TrEMBL databases and primary literature. Delineation of *B. pumilus* (ions at *m/z* 5271 and 6122) and *B. safensis* (ions at *m/z* 5288, 5568 and 6413) species were supported by a congruent characteristic protein pattern. Moreover, using a chemometric approach, the score plot created by partial least square discriminant analysis (PLSDA) of mass spectra demonstrated the presence of two individualized clusters, each one enclosing isolates belonging to a species-specific spectral group. The generated pool of species-specific proteins comprised mostly ribosomal and SASPs proteins. Therefore, in *B. pumilus* the specific ion at *m/z* 5271 was associated with a small acid-soluble spore protein (SASP O) or with 50S protein L35, whereas in *B. safensis* specific ions at *m/z* 5288 and 5568 were associated with SASP J and P, respectively, and an ion at *m/z* 6413 with 50S protein L32. Thus, the resulting unique protein profile combined with chemometric analysis, proved to be valuable tools for *B. pumilus* and *B. safensis* discrimination, allowing their reliable, reproducible and rapid identification.

## Introduction

*Bacillus pumilus* and *Bacillus safensis* represent one of the most significant and widespread terrestrial species within the *Bacillus pumilus* group [1], [2], [3], [4], [5]. A wide range of biotechnological and pharmaceutical applications has been attributed to these species, including human and animal probiotics [6] or phytosanitary-based products [7]. Moreover, they are important contaminant agents found in industrial settings, namely in food and/or pharmaceutical facilities, posing a serious problem to the quality assurance of these industrial segments [2], [4]. More rarely, *B. pumilus* isolates have also been involved in foodborne poisoning [8], [9] and in human infections including anthrax-like cutaneous lesions [10], [11], [12], [13].

Studying a collection of previously identified *B. pumilus* isolates we have realized the difficulty to their differentiation from closely related species based on phenotypic and biochemical characteristics and on 16S rRNA gene sequences [4]. Sequencing housekeeping genes, such as *gyrB* (β-subunit of DNA gyrase) and *rpoB* (β-subunit of RNA polymerase) has proven to be useful for taxonomic resolution of closely related species, including *Bacillus* species. Nevertheless, are already recognized its implementation difficulties in the routine of microbiology laboratories [4], [5].

Matrix-assisted laser desorption-ionization-time-of-flight mass spectrometry (MALDI-TOF-MS), is an accurate, fast, and affordable emerging technique that has been increasingly used for identification of bacteria at different taxonomical levels [14], [15]. As a proteomic approach, MALDI-TOF-MS relies on the reproducible detection of microbial protein patterns, which can be used for microbial identification by comparing experimental mass spectra with a library of known reference strains or by comparing identities of species-specific biomarkers [16], [17].

Using vegetative *B. pumilus* and *B. safensis* cells Farfour et al. [18] unsuccessfully attempted to discriminate these closely related species. Differentiation of these species using spore cells was proposed by Dickinson et al. [19], by MALDI-TOF-MS using a single ion at *m/z* 7620. Nevertheless, there is a need for further studies in order to assess the validity of this discriminatory tool. Moreover, Lash et al. [20], [21] suggested that bacterial vegetative cells when submitted to a protein enrichment process, could provide a more informative mass spectra pattern than that obtained with bacterial spores [20], [21].

In addition, the species-specific protein fingerprint constitutes a valuable approach for the assignment of species biomarkers, which usually comprise cell structure and housekeeping proteins [22]. In *Bacillus* spp., ribosomal proteins and small acid-soluble spore proteins (SASPs) have been reported as potential species-specific biomarkers [21]. Nevertheless and despite this successful characterization of *Bacillus cereus* group species [21], this was not applied to the members of the *B. pumilus* group.

In this work, through the implementation of a protocol that enhances proteins extraction [21] combined with chemometric tools, we assessed the potential of MALDI-TOF-MS fingerprinting to discriminate a comprehensive collection of *B. safensis* and *B. pumilus* isolates. Moreover, the tentative assignment of their species-specific protein biomarkers was also performed.

## Materials and Methods

### Isolates collection and identification

Five *B. pumilus* and twenty-two *B. safensis* isolates that were previously identified by phenotypic and genotypic (16S rRNA, *gyrB and rpoB* gene sequences) methods were studied [5], [23]. They comprised diverse pulsed-field gel electrophoresis (PFGE)-types and were recovered from different geographic terrestrial locations and sources, including food samples (Norway, Italy and Africa) (n = 4), plants (USA) (n = 2), gastropods (Portugal) (n = 3), health (n = 6) and cosmetic (n = 4) products (Portugal), and clean room environments from Mars Odyssey (USA) (n = 5). Additionally, type and reference strains, *B. safensis* FO-36b^T^, *B. pumilus* ATCC 7061^T^ and ATCC 14884 were included (Table 1).

**Table 1:** Origins of *Bacillus* spp. isolates (n = 27) included in this study.

Isolate	Origin	Year/Location	References
***Bacillus pumilus***			
Bp ATCC14884			Reference strain
Bp ATCC 7061^T^			Type Strain
Bp7		2005/Portugal^1^	
Bp11	Health products (n = 3)	2005/Portugal^1^	[37]
Bp15		2005/Portugal^1^	
***Bacillus safensis***			
Bs1		2004/Portugal^1^	
Bs2	Animals Gastropods (n = 3)	2005/Portugal^1^	
Bs3		2007/Portugal^1^	
Bs13		2005/Portugal^1^	
Bs16	Health products (n = 3)	2005/Portugal^1^	
Bs17		2005/Portugal^1^	[37]
Bs5		2002/Portugal^1^	
Bs18	Cosmetic products (n = 4)	2002/Portugal^1^	
Bs19		2002/Portugal^1^	
Bs27		2002/Portugal^1^	
Bs24		2004/Italy^2^	
Bs25	Foods/salame (n = 3)	2004/Italy^2^	[38]
Bs33		2004/Italy^2^	
Bs22	Plant Growth-PromotingRhizobacteria (PGPR) (n = 2)	1997/USA^3^	[39]
Bs23		1997/USA^3^	
Bs31	Food/beans (n = 1)	2003/Africa^4^	[40]
Bs FO-36b^T^	Clean-room/air particulate (n = 1)	1999/USA^5^	Type Strain, [2]
Bs35	Clean-room/floor (n = 1)	2001/USA^5^	
Bs36	Clean-room/cabinet top (n = 1)	2001/USA^5^	
Bs37	Clean-room/Mars Odyssey spacecraft surface (n = 2)	2001/USA^5^	[2]
Bs38			
Bs42	Clean-room/anteroom (n = 1)	2001/USA^5^	

T Type Strain.

1 Isolates obtained from the Quality Control Department (INFARMED), Lisbon, Portugal.

2 Isolates FEL 55 from salame felino, UNG22 from salame ungherese and MIL46 from salame milano obtained from the Istituto di Scienze delle Produzioni Alimentari (ISPA), Bari, Italy.

3 Isolates SE 49 (AP3) and SE 52 (AP7) from cucumber roots obtained from the Culture collection of the Department of Entomology and Plant Pathology, Auburn University, Alabama, USA.

4 Isolates Bs31 from African locust beans for *Soumbala production* obtained from Ouagadougou, Africa.

5 Isolates FO-36b, SAFN-027, SAFN-037, KL-052, 51-3C and 82-2C from spacecraft and assembly-facility surfaces obtained from California Institute of Technology, California, USA.

### Sample preparation

For MALDI-TOF-MS analysis, soluble proteins were extracted from a pure single colony of *Bacillus* spp. grown on LB agar (Merck, Darmstadt, Germany), which were subsequently cultured on the same medium, under aerobic conditions, for 24 h at 37°C. Bacterial cells were harvested by transferring three full loops (ca. 30 µl) from each agar plate into 20 µl of sterile water and were resuspended by vortexing. Bacterial inactivation was carried out applying the modified trifluoroacetic acid (TFA) (Sigma-Aldrich, St. Louis, MO) inactivation protocol [20], with some modifications previously established by Lash et al. (2008) [20] to improve the accuracy of the mass spectra. Briefly, 80 µl of pure TFA was added to 20 µl of each bacterial suspension. After gentle shaking (100 rpm) for 5 min at room temperature, the solution was centrifuged for 20 min at 28960 g^−1^ at 4°C. *Subsequently, the supernatant was* 10-fold *diluted with HPLC grade water* (*Millipore* Corp., Bedford, MA), *filtered throughout a 0.22 µm pore size filter (Millipore) and stored at −20°C until further analysis.*

### Mass spectrometry methods

Snapshots of different protein composition were detected and acquired by a MALDI-TOF/TOF mass spectrometer (4800 Plus MALDI TOF/TOF Analyzer, AB SCIEX, Framingham, MA), equipped with a 200-Hz frequency Nd:YAG laser, operating at a wavelength of 355 nm. Pulse ion extraction with a 1300 ns delay time was used for collecting spectra. Measurements were carried out in linear positive mode using an acceleration voltage of 19.4 kV (Grid 1), and a lens 1 voltage of 8 kV. Each spectrum was the accumulated sum of at least 2000 laser shots within the ion range at *m/z* 2000–12000, due to the good reproducibility of the spectral profile in this interval. All the spectra were externally calibrated using a commercial mixture of angiotensin I, ACTH (adrenocorticotropic hormone) and insulin (AB SCIEX, Framingham, MA) and analyzed with the Data Explorer software (Version 4.6, AB SCIEX, Framingham, MA).

For MALDI-TOF-MS experiments, 2 µl of the filtrated microbial dilution were mixed with 2 µl of a 12-mg/ml α-cyano-4-hydroxycinnamic acid (CHCA) (Sigma-Aldrich, St. Louis, MO) solution, prepared in 100% ACN (Acetonitrile, Sigma-Aldrich) and 0.3% TFA. 1 µl of the mixture was spotted onto a 123×81 mm stainless steel MALDI sample plate (Opti-TOF 384- Well insert, AB SCIEX, Framingham, MA) and allowed to dry at room temperature. For each isolate, two biological replicates (obtained from two different agar plates) were carried out, and the mean spectra were considered for the analysis. Mass spectra were analyzed with the Data Explorer software (v3.7, build 126, AB SCIEX, Framingham, MA). Ion masses were extracted from the raw experimental mass spectra that included all the ion peaks with a relative signal to noise (S/N) ratio intensity above 2.

### Chemometric methods

MALDI-TOF-MS spectra were mean-centred and analysed by partial least squares discriminant analysis (PLSDA) [24]. The PLSDA model were developed and validated based on a cross-validation strategy leave-one-out [25] where 70% of the strains were randomly selected to calibrate the model and 30% to test the model (the procedure was repeated 100 times). The PLSDA scores were the source for hierarchical cluster analysis (HCA). The purpose of HCA was the generation of dendrograms highlighting the association between isolates. Dendrograms were performed directly on unprocessed PLSDA scores using the Euclidean distance and the Median’s algorithm [26]. All chemometric models were performed in Matlab version 6.5 release 13 (MathWorks, Natick, MA) and the PLS Toolbox version 3.5 for Matlab (Eigenvector Research, Manson, WA).

### Biomarker identification

Intact protein masses derived from MS analysis were used to generate a pool of candidate’s proteins for the identification of specific markers. The selected distinct mass information was submitted to a web-based TagIdent software tool (http://web.expasy.org/tagident/) using 1% mass error for the taxonomic selections *B. pumilus*, *B. safensis* and *B. subtilis*. No restrictions on protein isoelectric point were used. This tool allowed the identification of proteins based on the experimental masses acquired by mass spectrometry using the information available at the UniProtKB/Swiss-Prot and UniProtKB/TrEMBL protein sequence databases. Moreover, ribosomal proteins of genome-sequenced type strains, including *B. pumilus* ATCC 7061^T^ available in the database developed by Hotta et al. [27] were also included for comparison. For ion peaks matching the theoretical molecular weights we took into consideration the “N-end rule” where N-terminal methionine is cleaved when the second amino acid residue had a small side-chain, according to the previous study [28].

## Results and Discussion

One of the current challenges in bacterial taxonomy is to integrate timely and accurate typing methods for a meaningful identification of microorganisms [14], which is particularly problematic for closely related species such as *B. pumilus* and *B. safensis* [2], [5]. Moreover, because of the medical, industrial and biotechnological relevance of these species, reliable, easy and rapid methodologies for their correct differentiation are needed. Discrimination of *B. pumilus* and *B. safensis* by molecular markers sequencing (e.g. *gyrB*), a time demanding and expensive methodology, which is not readily available for routine laboratories, was recently demonstrated [4], [5]. In fact, there are no comprehensive studies assessing its potential on *B. pumilus* and *B. safensis* discrimination despite the importance of the MALDI-TOF-MS application in bacterial differentiation. Moreover, combining accurate MALDI-TOF mass ion signals with the information available at a protein sequence database, such as UniProt, species-specific candidate protein biomarkers can be tentatively assigned, supporting the interest of this methodology for species identification.

### Sample preparation conditions

Bacterial species identification using MALDI-TOF-MS is based on mass profiles obtained from whole bacterial cell suspensions considering proteins with low mass weight (less than 20 kDa). Additionally, for MS applications it is imperative to define a standardized protocol, including the establishment of rigorous sample preparation and cultivation conditions, including culture medium, temperature and time. Therefore, an adequate number of ion masses should be obtained to allow the identification and discrimination of closely related bacterial species. Moreover, the definition of strict parameters for spectral data acquisition is also required.

The protein enrichment protocol, reported by Lash et al. [20], for *B. cereus* group members, using a combined TFA treatment, centrifugation and filtration steps, is of relevance in the case of *Bacillus* species, since it promoted an efficient extraction of soluble microbial proteins presented in the core spore and other morphological structures [20]. The described sample preparation procedure was successfully applied to *B. pumilus* and *B. safensis* isolates, clonally diverse and collected from different terrestrial origins [4], demonstrating its suitability to generate reproducible mass spectra data with a sufficient number of ion masses and reinforcing the potential for the successful application of the MS technique for their identification in routine laboratories.

### Mass spectrometry analysis

**Figure 1:**
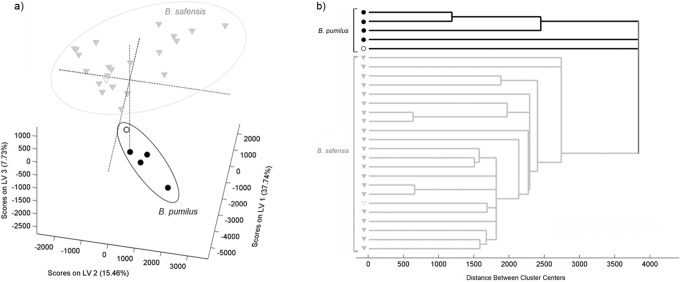
Average mass spectra obtained by MALDI-TOF-MS analysis of *B. pumilus* and *B. safensis* isolates. (a) *B. pumilus* (5 spectra) and (b) *B. safensis* (22 spectra) in the ion range at *m/z* 2000 to 12000. Spectra were obtained by averaging the respective experimental ion signals from all isolates.

The Figure 1 displayed the average mass spectra obtained from MALDI-TOF-MS analysis for the two *Bacillus* species under study: (a) *B. pumilus* (5 spectra) and (b) *B. safensis* (22 spectra), in the ion range at *m/z* 2000 to 12000. In fact, MALDI-TOF-MS profile analysis revealed the presence of species-specific ion signals. Table 2 compiled the characteristic ion masses for each spectral group and those presented in both species.

**Table 2:** Species-specific ion *m/z* values (average) of *B. pumilus* and *B. safensis* isolates.

	Experimental average ion *m/z* values^a^	
	*B. pumilus*	*B. safensis*
		3692.5
	3060 (BP1)^b^	3821.5
***B. pumilus***	3608.5 (BP2)	4305.5
	5271 (BP3)	5948.5
	6122 (BP4)	6704
		6793.5
		7415
	3692.5	
	3821.5	3396 (BS1)^b^
	4305.5	5288 (BS2)
***B. safensis***	5948.5	5568 (BS3)
	6704	6094 (BS4)
	6793.5	6413 (BS5)
	7415	

a average ion *m/z* values (±2Da).

b In brackets were named candidate species-specific ion masses.

As expected, similar fingerprints could be observed for isolates belonging to the same species, exhibiting several common ion peaks (Figure 1). For instance, the ions having *m/z* values at 3060, 3608.5, 5271 and 6122 were consistently observed in *B. pumilus* isolates, while the ions having the *m/z* values at 3396, 5288, 5568, 6094 and 6413 were related to *B. safensis*. Moreover, *B. pumilus* and *B. safensis* isolates demonstrated common ions at *m/z* 3692.5, 3821.5, 4305.5, 5948.5, 6704, 6793.5 and 7415 (Table 2), which could be considered characteristic ion peaks for both species. Therefore, the protein fingerprint similarities achieved among *B. pumilus* and *B. safensis* corroborated the closeness similarity previously verified among them [4], [5].

Interestingly, some spectral variability was observed within isolates belonging to the same spectral group, including the presence or absence of some ion peaks beyond those listed in table 2 (data not shown). This observation was not surprising, since we had previously demonstrated that both species comprised a clonally diverse population [4]. Therefore, the specific peptide profile probably reflects their evolution towards an adaptation to different niches [4].

Indeed, few studies have explored the potential of MALDI-TOF-MS to properly identify *B. pumilus* or *B. safensis* isolates [18], [19], [29], [30].

Farfour et al. attempted unsuccessfully to discriminate between *B. pumilus* and *B. safensis* vegetative cells by MALDI-TOF-MS using the Andromas database [18]. This discrepancy highlighted the need for the improvement and enlargement of this database and led eventually to the prior enrichment of the protein before MS analysis. Moreover, Böhme et al. [29], using *B. pumilus* type and reference strains (ATCC 7061^T^ and 14884) that were subjected to an extraction procedure previously to the MS analysis, suggested the presence of a series of ions at *m/z* 3620, 5297, 6617 and 7237, which were specific for this species, when compared with other *B. subtilis* group members (*B. subtilis*, *Bacillus amyloliquefaciens* and *Bacillus licheniformis*) and with members of *Bacillus cereus* group (*B. cereus*, *Bacillus megaterium* and *Bacillus thuringiensis*). In addition, the presence of this same series of ions at *m/z* 3620, 6617 and 7238 were also detected by Fernández-No et al. [30] in the same reference strains. Analysis of our MALDI-TOF-MS profiles did not reveal these ion peaks as *B. pumilus* species-specific discriminatory, when compared with *B. safensis*. Nevertheless, a closer inspection of the mass spectra of *B. pumilus* ATCC 7061^T^ and 14884, revealed the presence of ions having *m/z* values at 3621, 5290 and 6624, although the ion at *m/z* 7238 was not present. Indeed, the differences found in these *B. pumilus* profiles could be justified by the distinct growth culture medium used and the sample preparation procedure employed.

Finally, in other work, Dickinson et al. [19] presented MALDI-TOF-MS as a useful taxonomic tool for differentiating spores of *B. pumilus* and *B. safensis*. Results revealed the presence of two groups of characteristic ion peaks, comprising *B. pumilus* (ions at *m/z* 6860, 7230 and 9606) and *B. safensis* (ions at *m/z* 6860, 7230, 7620 and 9606). The authors claimed the presence of the additional ion at *m/z* 7620 in the spectra profile of *B. safensis* as a species-specific biomarker, allowing the discrimination of these two species. Nonetheless, analysis of our *B. safensis* spectral data (n = 22) did not reveal the presence of this ion peak, probably because the obtained MALDI-TOF-MS profiles were from vegetative cells. Additionally, the spectral profile obtained from spores seems to be more laborious and insufficient to discriminate appropriately among these closely related species, since spectral data with few number of ion peaks were generated. For these reasons, it was not possible to compare our study with the existing ones as we have used different cultural conditions and sample preparation procedures.

On the other hand, the species identification is limited to the bacterial species spectrum presented in a specific MS database. Therefore, bacterial identification is only possible inside the frame of bacterial reference spectrum of the database used. Indeed, few well-characterized *B. pumilus* isolates are available in public databases, as the SpectraBank (http://www.spectrabank.org), namely the type strain ATCC 7061^T^ and the reference strain ATCC 14884, and no *B. safensis* was yet included, which constrains its identification.

### Chemometric analysis

**Figure 2:**
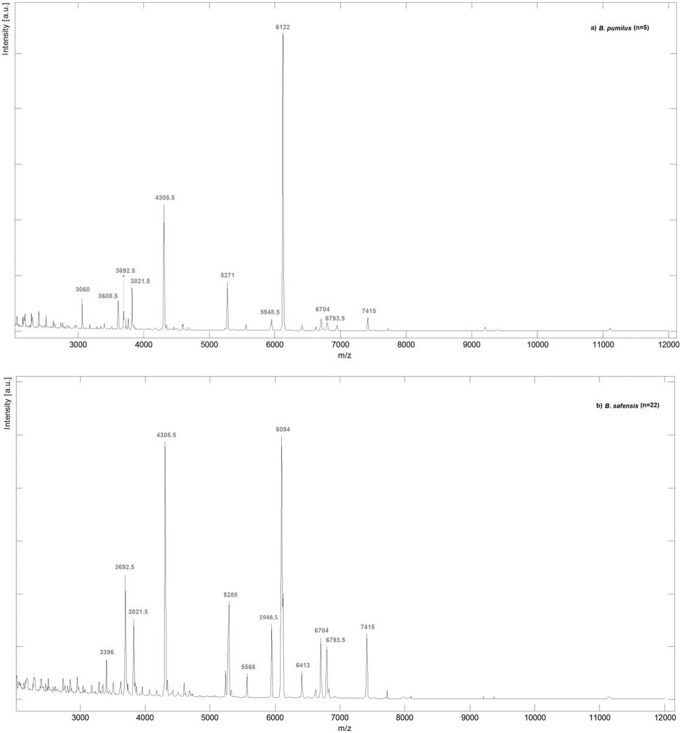
Score plot obtained by PLSDA regression model and the corresponding dendrogram. Score plot of the PLSDA regression model (a) and respective dendrogram (b), of *B. pumilus* and *B. safensis* isolates. Legend: • *B. pumilus isolates* and ▾ *B. safensis isolates*. Unfilled symbols correspond to the type strains of both species.

*B. pumilus* and *B. safensis* were clearly discriminated by a combined MALDI-TOF-MS and chemometric approach. The score plot generated by PLSDA of mass spectra of all isolates tested exhibited two individualized clusters, each one enclosing isolates belonging to a particular spectral group, which included reference and type strains (Fig. 2) of (a) *B. pumilus* and (b) *B. safensis*. The PLSDA scores were also presented as a dendrogram corroborating the species discrimination into two distinct clusters (Fig. 2). Moreover, this approach allowed the discrimination of the two *Bacillus* species with 100% of sensitivity and specificity.

Despite the recognized proficiency of chemometric tools for the analysis and identification of bacteria based on their fingerprint, in the case of *Bacillus* spp., this characterization was only previously applied in *B. cereus* group species [21]. Therefore, this is the first successful application of this approach considering members of *B. pumilus* group, stressing its relevance for discrimination among close related species.

### Candidate molecular biomarkers assignment

The possibility of biomarkers identification is one of the most valuable aspects of the mass spectrometric-based identification techniques, being this approach successfully applied to different bacterial species [21], [31], [32].

The assignment of 17 mass signals diagnostic ions formed in the MALDI-TOF-MS of *B. pumilus* and *B. safensis* represented the first consistent evidence of the relation between these ion *m/z* signals and the specific candidate protein sequences, which were presented in Table 3. Direct bacterial discrimination by means of MALDI-TOF-MS was hampered by the absence of consistent databases supported on sufficient identified biomarkers. Indeed, only two *B. pumilus* genomes with numerous proteins defined as unknown were available in UniProtKB/Swiss-Prot and UniProtKB/TrEMBL (*B. pumilus* SAFR-032 and ATCC 7061^T^) and no *B. safensis* were deposited, which hindered the candidate biomarkers identification. Therefore, this type of assignments can only be tentatively used to establish potential connections between protein sequences and ion *m/z* signals, and thus, should be prudently interpreted, although previously successfully applied in *B. cereus* group members [21].

**Table 3:** Overview of biomarkers tentative assignment of MALDI-TOF-MS mass signals of *B. pumilus* group species.

Specie(s)	Observed Mass (Da)	Predicted Mass (Da)	Error (%)	UniProt Accession ID	Protein Description	Peptide sequence	Organism^c^
	3060	NA	NA	Unassigned	NA		NA
	3608.5	NA	NA	Unassigned	NA		NA
		5270	0.02	A8FJG4	50S bRP subunit L34	MKRTFQPNNRKRSKVHGFRSRMSSKNGRLVLKRRRSKGRKKLSA	B. pumilus SAFR-032
	5271						
***B. pumilus***		5266.89	0.08	A8FDQ9	aSASP O	MTKRKANHVINGMNAAKSQGNGAGYIEDDQLVLTAEQRQNNKKRKKNQ	*B. pumilus* SAFR-032
		6114	0.2	C0H3U0	Uncharacterized membrane protein YyzG	MQTNRVILLAVMICLVSAITVFLLNGCKVDFLDIGGTIIGCFLGIFVVVRIQKKQS	*B. subtilis* subsp. *subtilis* str. 168
	6122						
		6126	0.08	P0C8M5	Transcriptional regulator SlrA	MKTHVKKDLDKGWHMLIQEARSIGLGIHDVRQFLESETASRKKNHKKTVRQD	*B. subtilis* subsp. *subtilis* str. 168
	3049	NA	NA	Unassigned	NA		NA
	3396	NA	NA	Unassigned	NA		NA
***B. safensis***	5288	5299.88*	0.2	A8FHD4	aSASP J	MSFFQKDKKAKSEKDHKQVDQLLEEASKELAGDPLQEAVQKKKNNDQ	*B. pumilus* SAFR-032
	5568	5544	0.5	A8FDQ8	aSASP P	MTNKNTGKDIRQNSPKEHQSGQPEPLSGSKKVKNRNHTRQKHNSHHDM	*B. pumilus* SAFR-032
	6094	NA	NA	Unassigned	NA		NA
	6413	6411.7*	0.03	A8FCW7	bRP subunit L32	MAVPFRRTSKMKKRLRRTHFKLQVPGMVACPECGEMKISHRVCKSCGTYKGKDVKSN	*B. pumilus* ATCC 7061^T^
	3692.5	3698	0.1	C0H3V1	UPF0752 membrane protein YczN	MSGYSNGGGYGGISSFALIVVLFILLIIVGTAFVGGF	*B. subtilis* subsp. *subtilis* str. 168
	3821.5	NA	NA	Unassigned	NA		NA
	4305.5	4305	0.01	A8F9A9	50S bRP subunit L36	MKVRPSVKPICEKCKVIRRKGKVMVICENPKHKQKQG	*B. pumilus* SAFR-032
***B. pumilus*** ** and ** ***B. safensis***	5948.5	5932	0.3	A8FF72	50S bRP subunit L33 2	MRVNITLACTECGERNYITKKNKRNNPDRVEFKKYCSRDKKQTVHRETK	*B. pumilus* SAFR-032
	6704	6691	0.3	C0H3Z1	Uncharacterized protein YjzG	MMKNGFAYKNGKLVNIFCGKEELYNELKAFLVKTFSINVKEVSRPSIYRRTKSKQLE	*B. subtilis* subsp. *subtilis* str. 168
	6793.5	6793*	0.01	B4AE40	50S bRP subunit L28	MARKCVITGRKTKAGNNRSHAMNSTKRTWGANLQKVRILVDGKPKRVYVSARALKSGKVERV	*B. pumilus* ATCC 7061^T^
	7415	7410.8*	0.08	B4AM90	50S bRP subunit L35	MPKMKTHRGSAKRFKKTGSGKLKRSHAYTSHLFANKSTKQKRKLRKSAIVSAGDFKRIKQQLANIK	*B. pumilus* ATCC 7061^T^

Protein identity was determined by the TagIdent software and compared with ribosomal subunit proteins developed by Hotta et al. [23] described in Materials and Methods section.

a SASP - Small, acid-soluble spore protein.

b RP – Ribosomal protein.

c Organism – bacterial strain where the protein was described.

NA – not applicable.

* Predicted molecular weight (Mw) proposed by Hotta et al. [27], considering “N-end rule” [28] where N-terminal methionine is cleaved from specific penultimate amino acid residues such as glycine, alanine, serine, proline, valine, threonine and cysteine.

We found evidences of *B. pumilus* and *B. safensis* specific biomarkers, associated with a series of ions at *m/z* 4305.5, 5948.5, 6793.5 and 7415, which was attributed to the 50S ribosomal subunits proteins, respectively, L36, L33, L28 and L35 of B. pumilus SAFR-032 (correspondent amino acidic sequences were also presented in Table 3). Moreover, the remaining ion detected at *m/z* of 3821.5 was not assigned, and two showed correspondences with membrane proteins of *B. subtilis* subsp. *subtilis* str 168, the YczN and YjzG at *m/z* of 3692.5 and 6704, respectively.

The tentative assignment of *B. pumilus* specific biomarkers revealed the possible correspondence of the diagnostic ion at *m/z* 5271 with the 50S ribosomal subunit protein L34 or with the SASP O. In addition, the characteristic ion at *m/z* 6122 was diagnostic for either the uncharacterized membrane protein (YyzG) and/or the transcriptional regulator – SlrA of *B. subtilis* subsp. *subtilis* str 168. Additionally, the characteristic ions at *m/z* 3060 and 3608.5 were not possible to assign.

**Figure 3:**
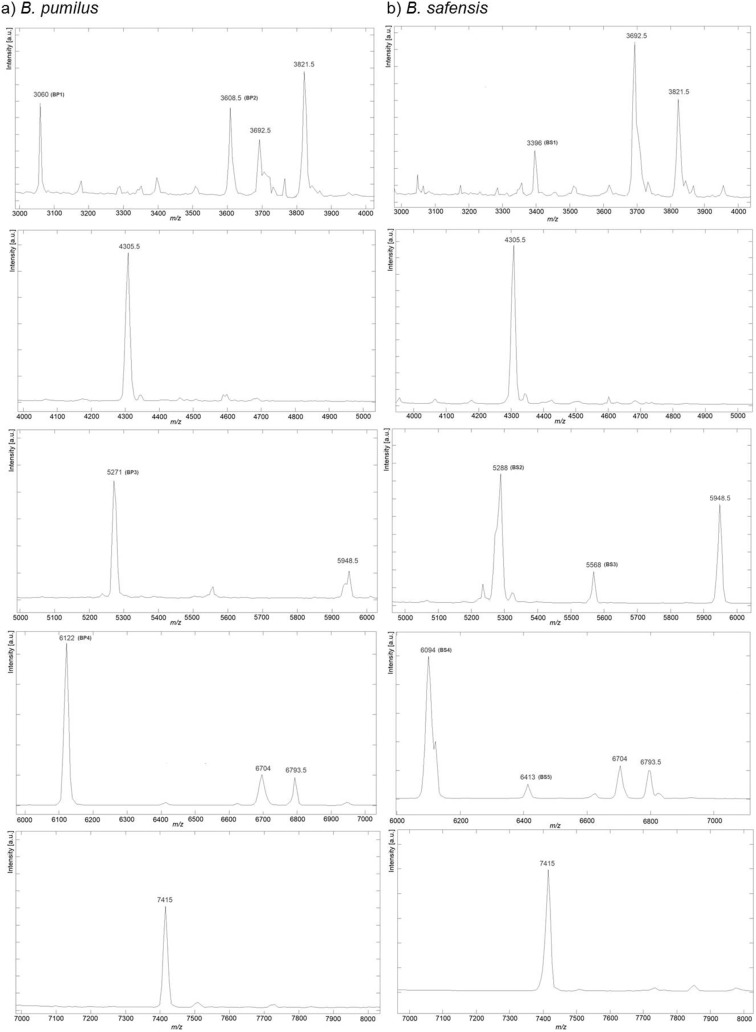
MS patterns of the candidate species-specific ion peaks (*m/z*) using MALDI-TOF-MS in linear mode. a) *B. pumilus* (average of 5 spectra) and b) *B. safensis* (average of 22 spectra). In brackets were named candidate species-specific ion peaks.

Concerning *B. safensis* specific ions at *m/z* 5288, 5568 and 6413, potentially corresponding with two specific SASPs (SASP J and SASP P) and a 50S ribosomal subunit protein L32 were also found in *B. pumilus* SAFR-032. The remaining ion *m/z* peaks detected were not possible to designate. Therefore, the proposed characteristic biomarkers, which could be used to differentiate between *B. pumilus* and *B. safensis* are summarized in Table 4. Moreover, these differentiating series of ions were shown in Figure 3, which outlined the representative MALDI-TOF-MS biomarkers established for *B. pumilus* and *B. safensis*.

**Table 4:** Candidate species-specific biomarkers assignments of *B. pumilus* and *B.safensis*.

	Species	
Ion mass (*m/z*)	*B. pumilus*	*B. safensis*
**5271**	+	−
**5288**	−	+
**5568**	−	+
**6122**	+	−
**6413**	−	+

Ion masses are presented as *m/z* values. The presence/absence of an ion peak in each spectral group is represented by +/−, respectively.

In fact, ribosomal proteins and small, acid-soluble spore proteins (SASPs) have been suggested to be responsible for many ion masses detected by MALDI-TOF-MS profiles [21]. Since up to 21% of the overall cellular protein content is ribosomal and because ribosomal proteins are part of the cellular translational machinery constitutively expressed in vegetative cells, they constitute a stable ensemble of protein biomarkers suitable for use by fingerprinting techniques [33]. Moreover SASPs, a group of species-specific proteins present in large amounts in the core region of *Bacillus* endospores, have been also proposed as biomarkers for rapid differentiation and identification of *Bacillus* spp. using mass spectrometry approaches [34], [35], [36].

Our results also suggested that ribosomal and spore proteins constituted most of the *B. pumilus* and *B. safensis* biomarkers. A more detailed analysis could be carried out with MS/MS peptide fragmentation of the specific proteins assigned and subsequent comparison in protein databases or even with MS/MS peptide *de novo* sequencing. Nevertheless, within the context of the present work, which aimed to establish a MALDI-TOF-MS fingerprint classification for *B. pumilus* and *B. safensis,* these results may be beneficial and improve further accuracy of MS-based detection methods in identifying these species.

## Conclusion

MALDI-TOF-MS profiles combined with chemometric analysis (PLSDA) proved to be valuable tools for discrimination of *B. pumilus* and *B. safensis*, allowing its rapid identification. These high throughput approaches should be promptly considered for *Bacillus* species identification due to the inaccuracy of conventional techniques in the identification of closely related species of this genus. In this sense, it is imperative to standardize a sample preparation protocol, which should include a protein extraction and enrichment step, to provide informative and reproducible mass spectra. Moreover, tentative assignment of *B. pumilus* and *B. safensis* protein biomarkers suggested that most of them are ribosomal and spore proteins.
