# Correction: Differentiation of *Bacillus pumilus* and *Bacillus safensis* Using MALDI-TOF-MS

**DOI:** 10.1371/journal.pone.0116426

**Published:** 2014-12-19

**Authors:** The PLOS ONE Staff

Affiliation 4 and affiliation 5 are incorrectly switched. The affiliation for João Lopes should be: Departamento de Tecnologia Farmacêutica, Faculdade Farmácia, Universidade de Lisboa, Lisboa, Portugal. The affiliation for Manuela E. Pintado should be: CBQF, Centro de Biotecnologia e Química Fina, Escola Superior de Biotecnologia, Universidade Católica Portuguesa, Porto, Portugal.

The images for Figures 1 and 2 are incorrectly switched. The image that appears as Figure 1 should be Figure 2, and the image that appears as Figure 2 should be Figure 1. The figure legends appear in the correct order. Please view the correct images for Figure 1 and Figure 2 here.

**Figure 1:**
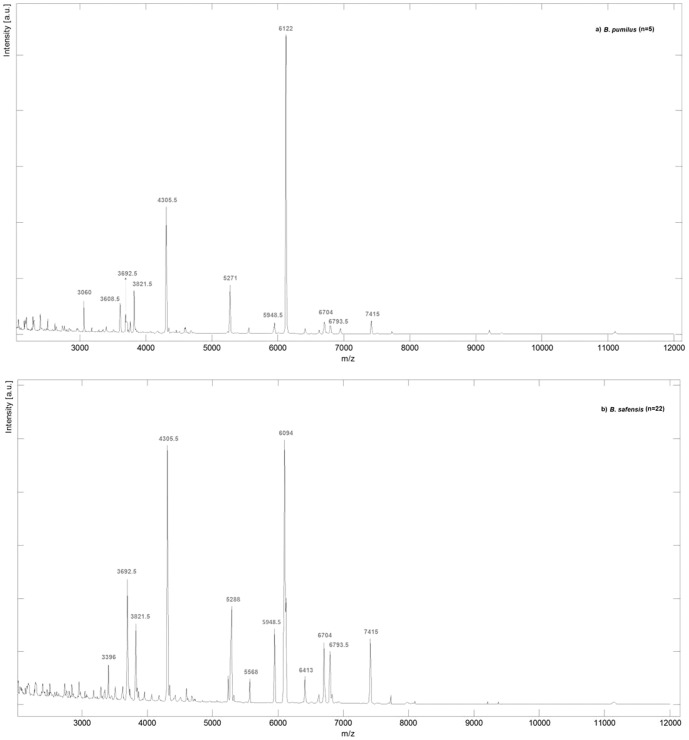
Average mass spectra obtained by MALDI-TOF-MS analysis of *B. pumilus* and *B. safensis* isolates. (a) *B. pumilus* (5 spectra) and (b) *B. safensis* (22 spectra) in the ion range at*m/z* 2000 to 12000. Spectra were obtained by averaging the respective experimental ion signals from all isolates.

**Figure 2:**
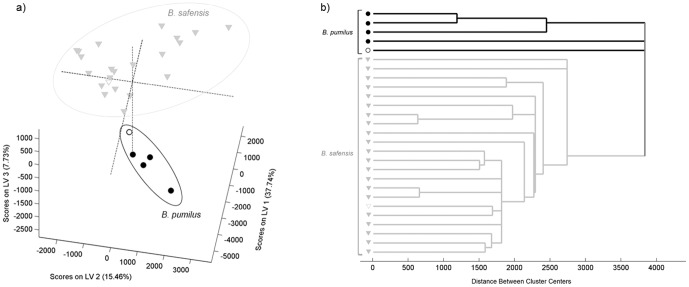
Score plot obtained by PLSDA regression model and the corresponding dendrogram. Score plot of the PLSDA regression model (a) and respective dendrogram (b), of*B. pumilus* and *B. safensis* isolates. Legend: • *B. pumilus isolates* and ▾ *B. safensis isolates*. Unfilled symbols correspond to the type strains of both species.
